# Association of CD8 T cell apoptosis and *EGFR* mutation in non‐small lung cancer patients

**DOI:** 10.1111/1759-7714.13504

**Published:** 2020-06-04

**Authors:** Chao Zhao, Chunxia Su, Xuefei Li, Caicun Zhou

**Affiliations:** ^1^ Department of Lung Cancer and Immunology, Tongji University Affiliated Shanghai Pulmonary Hospital Tongji University School of Medicine Shanghai China; ^2^ Department of Medical Oncology, Tongji University Affiliated Shanghai Pulmonary Hospital Tongji University School of Medicine Shanghai China

**Keywords:** Apoptosis, immunotherapy, lung cancer, tumor infiltrating lymphocytes

## Abstract

**Background:**

The abundance of tumor infiltrating CD8 T cells is an important parameter for antitumor effect of PD‐1/PD‐L1 immune checkpoint inhibitors, which is less in epidermal growth factor receptor (*EGFR*) mutation than wild‐type non‐small cell lung cancer (NSCLC). The mechanism still requires further study.

**Methods:**

In total 190 surgical lung adenocarcinoma samples were included. *EGFR* mutation was detected using amplification‐refractory mutation system. CD8 T cells and apoptosis were assessed by immunohistochemistry and immunofluorescence staining in tumor samples. Exosomes extracted from lung cancer cell lines with and without *EGFR* mutation were used to test the function of promoting apoptosis in vitro.

**Results:**

The ratio of CD8 tumor infiltration lymphocytes was significantly lower in *EGFR*‐mutant than in wild‐type patients (*P* = 0.026). A higher ratio of apoptosis was also prone to occur in *EGFR*‐mutant patients (*P* = 0.035). The distribution of apoptosis was not statistically associated with the ratio of CD8 TILs. An in vitro experiment indicated that exosomes secreted by *EGFR*‐mutant non‐small cell lung cancer cell lines PC9 and HCC827 were more capable of promoting CD8 T cell apoptosis than *EGFR* wild‐type cell lines H1299 and SK‐MES‐1 (*P* = 0.007 and *P* = 0.010, respectively).

**Conclusions:**

Non‐small cell lung cancer *EGFR* mutation could promote CD8 T cell apoptosis more than wild‐type. Inhibiting CD8 + TILs apoptosis may strengthen immunotherapy effects in *EGFR*‐mutant NSCLC patients.

## Background

Immunotherapy targeting programmed cell death receptor 1/ligand 1 (PD‐1/PD‐L1) has been shown to exhibit good effect and durable response in epidermal growth factor receptor (*EGFR*) and anaplastic lymphoma kinase (*ALK*) wild‐type non‐small cell lung cancer (NSCLC) patients.^1^, ^2^, ^3^, ^4^, ^5^, ^6^, ^7^ However, in *EGFR*‐mutant NSCLC patients, the response rate has been reported to be very low.^8^, ^9^ Compared with *EGFR* wild‐type patients, *EGFR*‐mutant patients do not appear to benefit more from immunotherapy than chemotherapy.^10^, ^11^ The underlying mechanisms therefore still require further study.

A retrospective study showed that CD8 + T‐cell abundance was the most predictive parameter of the response to anti‐PD‐1/PD‐L1 therapy across many cancer types,^12^ and the ratio of CD8 + TILs was significantly lower in *EGFR*‐mutant than in wild‐type patients.^10^, ^13^ Increasing the number of CD8 + TILs may be a promising strategy to enhance the immunotherapy effect for *EGFR* mutation in NSCLC patients.

Zhu *et al*. reviewed former studies, and found that TILs frequently undergo apoptosis induced by tumor cells,^14^ which may influence the abundance. Horton *et al*. showed that tumor could specifically induce CD8 + TILs apoptosis, and reducing CD8 + TILs apoptosis could boost tumor control when combined with immutherapy.^15^ In immunotherapy, CD8+ T cell apoptosis could also be a key point which impedes the effect of one immune inhibitor on another.^16^

Exosomes are cell released extracellular vesicles with a size range of 40–160 nm in diameter, and play important roles in regulating the tumor microenvironment.^17^ PD‐L1 positive NSCLC exosomes have been reported to induce CD8 + T cell apoptosis.^18^ Exosomes expressing FasL have also been reported to mediate CD8 + T cell apoptosis.^19^ Whether exosomes of *EGFR*‐mutant and wild‐type NSCLC have a different ability to promote CD8 + T cell apoptosis has not been previously reported.

From the above studies, we presume that the apoptosis caused the difference of CD8 + TILs abundance between *EGFR*‐mutant and wild‐type NSCLC. In this study, lung adenocarcinoma samples and peripheral blood were used to detect apoptosis of CD8 + T cells, and we hoped that the results would provide indications for current immunotherapy.

## Methods

### Patients

From January 2017 to December 2018, 190 formalin‐fixed paraffin‐embedded surgical lung adenocarcinoma samples in Shanghai Pulmonary Hospital were included. All patients consented to *EGFR* gene detection. Disease‐free survival (DFS) and overall survival (OS) were followed‐up until November 2019. The study was approved by the Ethics Committee of Shanghai Pulmonary Hospital (K19‐066Y).

### Gene mutation detection

*EGFR* mutation detection was conducted using the Amplification‐Refractory Mutation System. FFPE DNA extraction kit (Amoy Diagnostics, Xiamen, China) was used to extract tumor DNA. *EGFR* mutation was detected by *EGFR* 29 Mutations Detection Kit (Amoy Diagnostics, Xiamen, China) using 80 ng DNA. The procedures were followed‐up as described in the protocol. PCR was performed on a Stratagene Mx3000P cycler (Agilent, Santa Clara, CA, USA) using the following program: Five minutes at 95°C (one cycle); 25 seconds at 95°C, 20 seconds at 64°C, and 20 seconds at 72°C (15 cycles); 25 seconds at 93°C, 35 seconds at 60°C, and 20 seconds at 72°C (31 cycles). The results were determined as described in the protocol.

### Immunohistochemistry and immunofluorescence staining

CD8 immunohistochemistry antibody (cat no.ab4055, Abcam) was used to stain TILs in tumor samples. Brown or yellow‐brown staining of tumor infiltrating cells was defined as positive. CD8 TILs was grouped using a cutoff value of 5% similarly as reported in other studies.^13^, ^20^ Apoptosis was stained using TUNEL apoptosis staining kit (cat no.11684817910, Roche), and low, medium and high levels were determined as <1%, 1–5% and ≥5%. CD8 and TUNEL immunofluorescence costaining was conducted in 10 randomly selected tumor samples to show the condition of CD8 + T cell apoptosis.

### CD8 + T cell apoptosis detection

Peripheral blood samples were collected from 11 healthy volunteers, and utilized for peripheral blood mononuclear cells (PBMCs) separation in two hours using Ficoll‐Paque PREMIUM (cat no.17544203, GE Healthcare). PBMCs were placed on a 12‐well plate, and cultured in DMEM medium supplemented with 10% fetal bovine serum, 100 U/mL penicillin, and 100 mg/mL streptomycin for 4–6 hours. Then, 30 μg exosomes extracted from cell lines culture supernatants were added to the medium, and cultured for 48 hours. Finally, CD3 (557 832, BD Pharmingen), CD8 (555 366, BD Pharmingen) and Annexin‐V (88 800 774, eBioscience) were stained and analyzed using flow cytometry.

### Exosome extraction and detection

The culture supernatants of PC9, HCC827, H1299 and SK‐MES‐1 cell lines were used to extract exosomes by total exosome isolation reagent (cat no.4478359, Invitrogen). Briefly, the supernatants were centrifuged at 2000 rpm for 30 minutes. The debris was discarded, and the supernatants were added to half the volume of the isolation reagent, and recentrifuged at 10 000 *g* for 60 minutes at 4°C. The precipitates were dissolved in 50–100 μL PBS buffer and stored at −80°C for future use.

### Statistical analysis

All statistical analyses were performed using SPSS v.20 software (SPSS Inc., Chicago, IL, USA). Comparisons of clinicopathological features between different CD8 stainings were evaluated by Pearson Chi‐square test or Fisher's exact test. Mann‐Whitney U test was used to analyze the features among different apoptosis levels. A paired *t*‐test was used for exosome‐induced CD8 + T cell apoptosis analysis. The two‐sided significance level was set at *P* < 0.05.

## Results

### Ratio of CD8 TILs in lung cancer patients

CD8 was stained in 161 tumor samples, and grouped as high and low ratio using cutoff value of 5% (Figure S1). The clinicopathological features between high and low ratio CD8 TILs were investigated in the study (Table 1). *EGFR* mutation samples had a lower ratio of CD8 expression than wild‐type (*P* = 0.026), and there was no significant difference in other clinicopathological features.

**Table 1:** Clinicopathological features of CD8 staining surgical lung adenocarcinoma samples

	CD8		
	Low N (%)	High N (%)	*P*‐value
Sex			
Female	49 (58.3)	42 (54.5)	0.628
Male	35 (41.7)	35 (45.5)	
Age			
<65	45 (53.6)	39 (50.6)	0.711
≥65	39 (46.4)	38(49.4)	
Smoking status			
Never	61 (72.6)	54 (70.1)	0.727
Light/smoker	23 (27.4)	23 (29.9)	
*EGFR* status			
Mutation	55 (65.5)	37 (48.1)	0.026
Wild‐type	29 (34.5)	40 (51.9)	
Disease stage			
I–II	75 (89.3)	66 (85.7)	0.493
III–IV	9 (10.7)	11 (14.3)	
T			
T1	78 (92.9)	71 (92.2)	0.875
Others	6 (7.1)	6 (7.8)	
N			
N0	78 (92.9)	70 (90.9)	0.650
Others	6 (7.1)	7 (9.1)	
M			
M0	75 (89.3)	66 (85.7)	0.493
Others	9 (10.7)	11 (14.3)	

### Ratio of apoptosis in lung cancer patients

**Figure 1:**
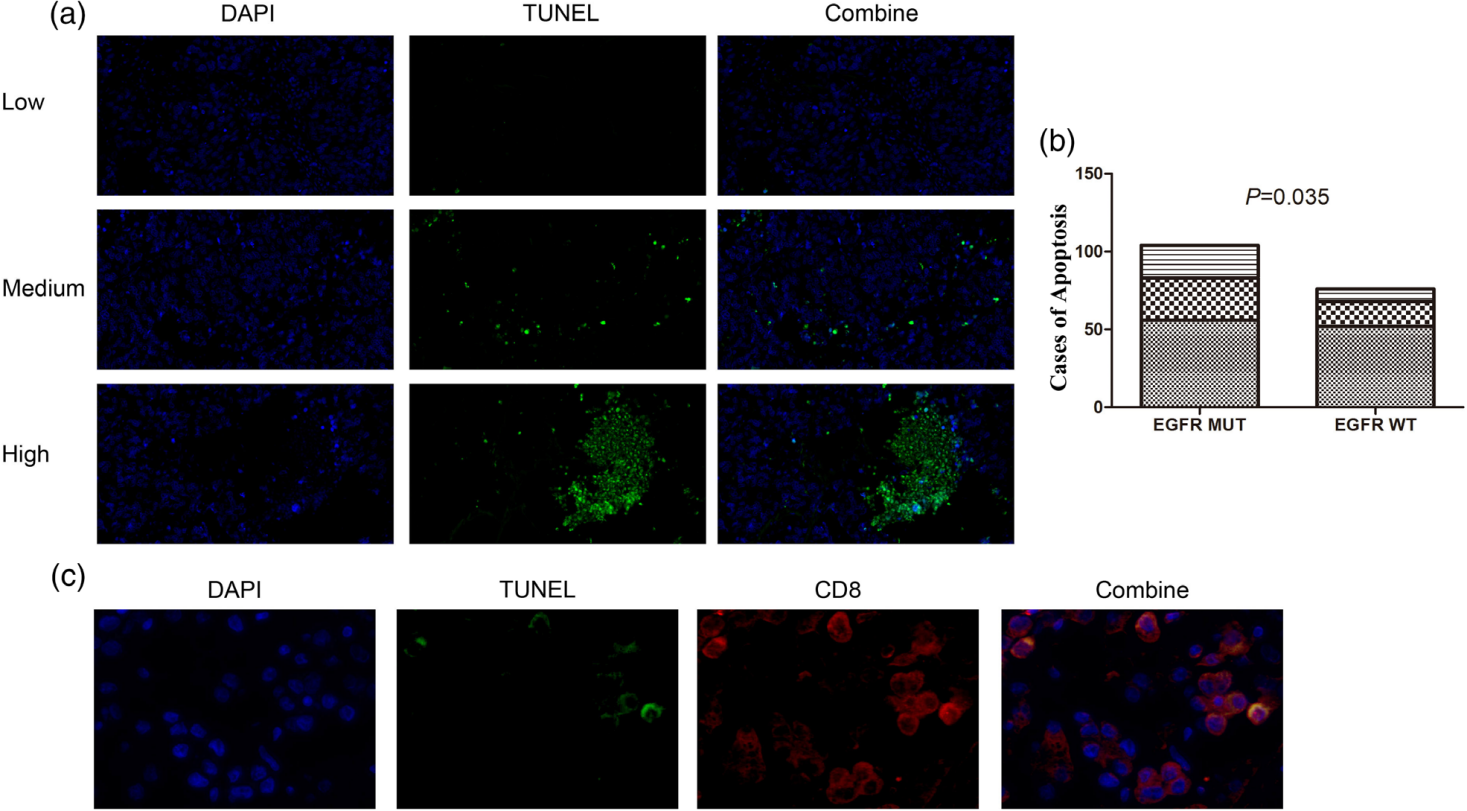
Apoptosis in lung adenocarcinoma samples. (**a**) DAPI and TUNEL were stained in tumor samples. Low refers to the ratio of apoptosis less than 1%; medium refers to 1%–5%; high refers to above 5%. (**b**) The distribution of apoptosis ratio was compared between *EGFR* mutation and wild‐type samples (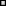) Low, (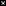) Medium, and (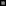) High. (**c**) CD8 and TUNEL immunofluorescence costaining was conducted in tumor samples to show CD8+ T cell apoptosis.

Total cell apoptosis was stained in the 190 samples. As more than half the samples had a low ratio of apoptosis, which was less than 1%, we further used a 5% cutoff value to classify the higher ratio of apoptosis. A higher level of apoptosis was prone to occur in patients harboring an *EGFR* mutation (*P* = 0.035) (Fig 1a,b). CD8 and TUNEL immunofluorescence costaining showed that CD8 + T cell underwent apoptosis (Fig 1c). However, as the sample number was small (five *EGFR* mutation and five wild‐type), we did not further analyze the association between the ratio of CD8 + T cell apoptosis and *EGFR* mutation status. We analyzed the association between *EGFR* subtypes and apoptosis, and found no significant difference between *EGFR* 19del and L858R, as well as *EGFR* common mutations (19del/L858R) and rare mutations (Figure S2A,B). The distribution of apoptosis on other clinical features was also not significantly different, except on disease stage (Figure S2C–F). Multivariate analysis showed that disease stage, but not *EGFR* mutation status, was significantly associated with the ratio of apoptosis (*P* = 0.003, Table S1). We will, therefore, study further with in vitro experiements whether *EGFR* mutation has a stronger ability than wild‐type.

### Association between ratio of CD8 TILs and apoptosis

**Figure 2:**
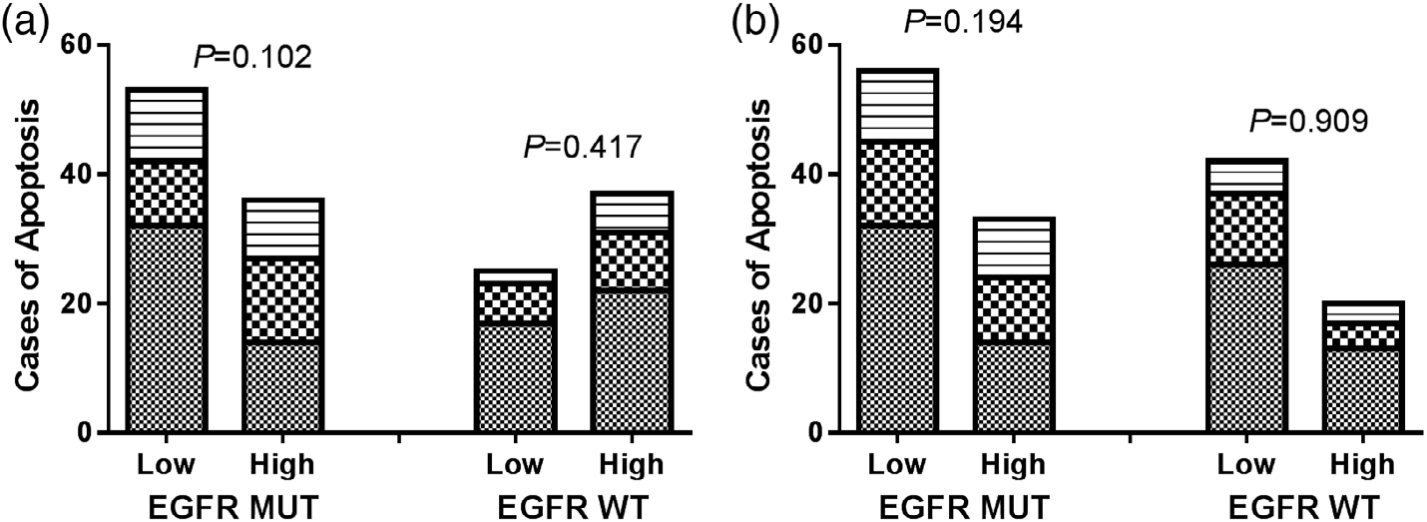
The association between the ratio of CD8 and apoptosis was analyzed in *EGFR* mutation and wild‐type samples. Low, medium and high levels of apoptosis were determined as <1%, 1–5% and ≥5%. The cutoff value of CD8 ratio was 5% (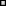) High, (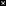) Medium, and (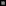) Low (**a**) and 10% (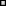) High, (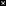) Medium, and (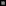) Low (**b**).

We investigated whether there was an association between CD8 TILs ratio and apoptosis in *EGFR* mutation and wild‐type patients (Fig 2). However, in the *EGFR* mutation as well as wild‐type groups, there was no significant difference (*P* = 0.102 and *P* = 0.417, respectively). Another cutoff value was used, but also showed no significant difference between the patients.

### Exosomes from *EGFR* mutation cell lines induced CD8 + T cell apoptosis

**Figure 3:**
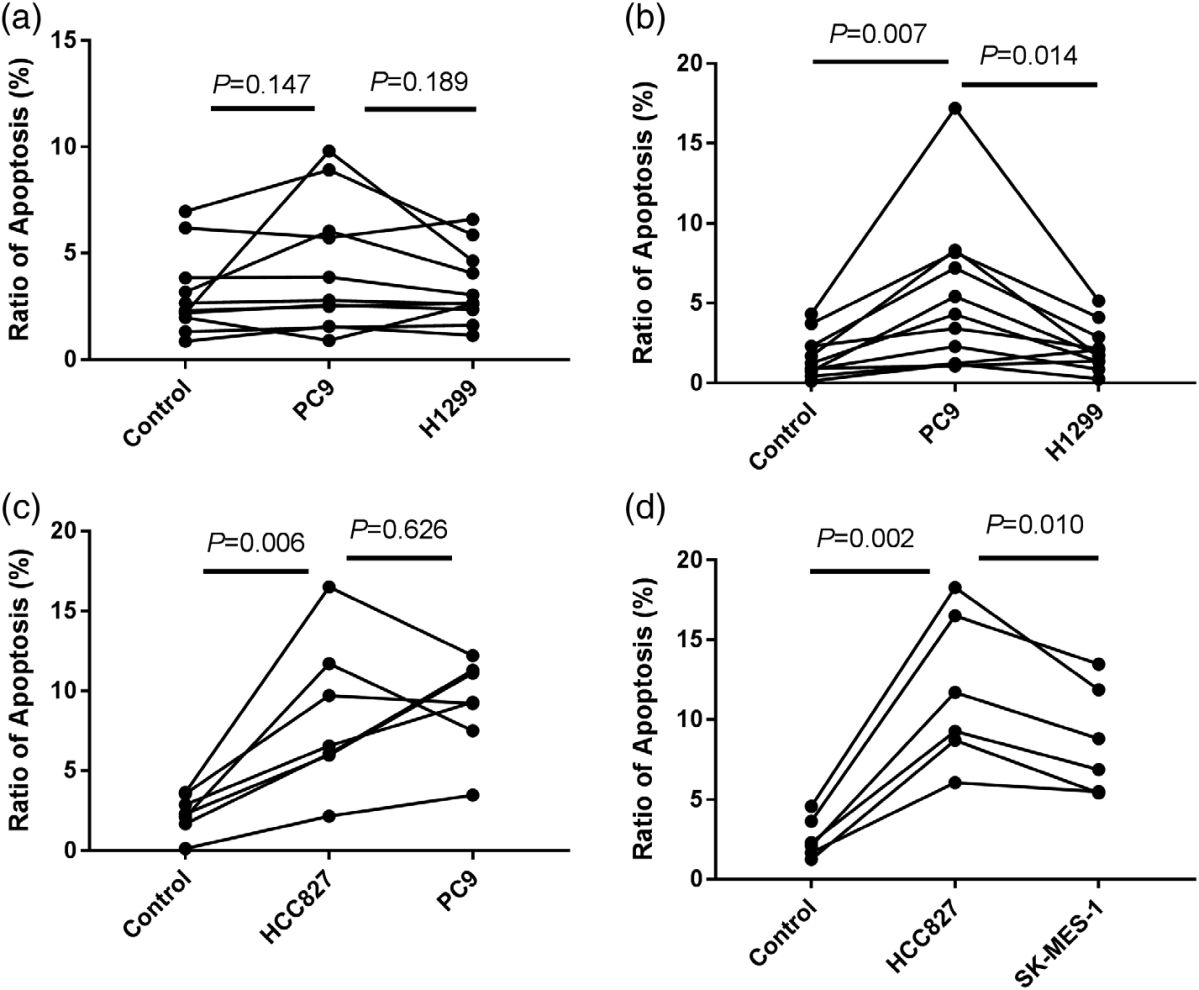
The function of exosomes in promoting apoptosis. Exosomes from *EGFR* mutation cell lines PC9 and HCC827, and wild‐type cell lines H1299 and SK‐MES‐1 were extracted and used to treat PBMCs from healthy volunteers. The apoptosis ratio of CD3+ PBMCs (**a**) and CD3 + CD8+ cells (**b**, **c**, **d**) were compared, respectively. Control: PBMCs without exosome treatment; PC9, HCC827, H1299, SK‐MES‐1: PBMCs treated with 30μg PC9, HCC827, H1299, SK‐MES‐1 cell exosomes for 24 hours.

To further explore whether *EGFR* mutation and wild‐type had different effects on CD8 T cell apoptosis, exosomes from *EGFR* mutation cell line PC9 and wild‐type cell line H1299 were used to treat cultured PBMC (Fig 3). The results showed that although there was no significant difference in apoptosis of all the treated cells between *EGFR* mutation and wild‐type groups, PC9 cell exosomes could promote CD8 T cell apoptosis, and the effect was stronger than that from H1299 (*P* = 0.007). We also found that PC9 exosomes possessed a similar ability to promote CD8 + T cell apoptosis with *EGFR* mutation cell line HCC827 exosomes (*P* = 0.626, Fig 3c), and HCC827 exosomes had a stronger ability to promote CD8 + T cell apoptosis than the *EGFR* wild‐type cell line SK‐MES‐1 (*P* = 0.010, Fig 3d).

### Patient survival

Until the end of the follow‐up date, 161 patients had no disease recurrence or metastasis, DFS data was obtained in four patients, and 25 patients were lost to follow‐up. Of the four patients, two were harboring *EGFR* mutation and two were harboring *EGFR* wild‐type (Table 2). Three of the four patients had an apoptosis ratio greater than 5%. However, the treatment was complicated in these patients, and it is therefore unreasonable to speculate the function of apoptosis to patient prognosis based only on DFS data. More survival data are needed in order to carry out further analyses.

**Table 2:** Characteristics of patients obtained DFS data

No.	Sex	Age	Smoking status	Disease stage	*EGFR* status	Treatment	CD8 ratio	Apoptosis ratio	DFS (month)	OS (month)
1	M	69	Yes	IV	19del	TKI†	Low	>5%	18.0	NA
2	F	71	None	I	WT	NA	Low	>5%	9.5	NA
3	F	54	None	IV	L861Q	TKI	High	>5%	8.0	NA
4	M	68	Yes	IV	WT	Chemo	Low	<1%	6.6	NA

TKI, tyrosine kinase inhibitors; Chemo, chemotherapy.

## Discussion

Immunotherapy has been widely used in *EGFR* and *ALK* wild‐type NSCLC patients; however, in *EGFR* mutation or *ALK* rearrangement patients, the effect is unsatisfactory. The mechanism behind the mystery therefore still needs to be determined.

To date, there are three main factors to explain this issue: TILs, tumor mutation burden (TMB), and PD‐L1 expression. The abundance of CD8 + TILs has been reported to be a better parameter than the other two factors to predict the response to anti‐PD‐1/PD‐L1 therapy across many cancer types.^12^ We and others have reported that CD8 + TILs have been found to be relatively lower in *EGFR*‐mutant than in wild‐type patients.^10^, ^13^ In this study, we aimed to explore what induces the difference between the two patient groups. Recently, the mechanism of apoptosis in TILs has been reviewed, but the study was mainly focused on the Fas‐FasL pathway.^14^ A study in a melanoma model showed that CD8 + TILs had high rates of apoptosis and preventing apoptosis could improve tumor control.^15^ These studies indicate that CD8 + TILs could undergo apoptosis in a tumor microenvironment, and inhibiting apoptosis may boost the immunotherapy effect. In this study, apoptosis was stained in tumor tissues and the results showed that *EGFR* mutation samples had a higher apoptosis ratio of tumor infiltrating cells than wild‐type. Multivariate analysis showed that *EGFR* mutation status was not significantly associated with CD8 + T cell apoptosis. However, based on the previous results in the study, cell line experiments in vitro could be used to further detect whether *EGFR* mutation could promote CD8 + T cell apoptosis compared with wild‐type. In the future, we will also collect more advanced stage samples to confirm whether disease stage is an independent factor to influence CD8 + T cell apoptosis.

Next, we analyzed the association between the ratio of CD8 TILs and apoptosis in *EGFR*‐mutant or wild‐type patients; however, no significant difference was found. As far as we know, there may be two potential reasons. First, the ratio of CD8 TILs had no association with the ratio of apoptosis. Second, there was an association between them, but it was concealed by apoptosis of other cells. To further test the promoting apoptosis function of *EGFR* mutation and wild‐type NSCLC to CD8 + T cells, exosomes were extracted from cell lines and used to treat CD8 + T cells in vitro. Exosomes are a type of extracellular vesicle secreted by cell types that mediate intercellular communication, and also play important roles in immunotherapy.^21^ The results suggested that exosomes could promote CD8 + T cell apoptosis, and the function was stronger in *EGFR*‐mutant NSCLC than wild‐type. Based on the results, more questions were raised, such as which content induces apoptosis, which subtype of CD8 TILs undergoes apotosis, or are there other types of cell that experience apoptosis? It has been reported that PD‐L1 expression on exosomes of NSCLC patients could promote CD8 + T cell apoptosis, rather than inhibit proliferation.^18^ As exosomes are composed of many proteins, RNA and lipids, whether there is another factor, especially that is different between *EGFR* mutation and wild‐type exosomes which induces apoptosis, should be studied in the future. The abundance of CD8 + CD39 + T cells was reported to be a good parameter for immunotherapy, and was less in *EGFR*‐mutant than wild‐type NSCLC patients.^22^ Whether this kind of cell undergo apoptosis more in *EGFR* mutation than wild‐type NSCLC patients also need to be studied further.

There are limitations in this study. First, the apoptosis was stained on all the cells to overview the panorama. However, limited by the economic cost, the costaining of CD8 and apoptosis was only conducted on some of the samples to show the state of CD8 + TIL apoptosis. Costaining would be helpful to compare CD8 TILs apoptosis between *EGFR* mutation and wild‐type samples, which will be implemented in more samples in future studies. Second, the CD8 + T cells used in our study were from the PBMC of healthy volunteers, and therefore were not fully representative of those in the tumor microenvironment of NSCLC patients. CD8 TILs and their apoptosis could be detected in surgical fresh tumor tissues as well as biopsy tissues to validate the results. Third, the patients were mostly at an early stage, the survival data were not mature and also not fully analyzed in the study. Survival data needs further follow‐up to investigate the influence of different ratios of CD8 TILs and apoptosis to survival.

In conclusion, our study determined that there was more apoptosis in *EGFR*‐mutant lung adenocarcinoma patients, and exosomes of *EGFR* mutation cell lines could promote CD8 + T cell apoptosis. Inhibiting CD8 + TILs apoptosis may be a feasible strategy to strengthen the immunotherapy effect.

## Disclosure

The authors declare that they have no competing interests.

## Supporting information


**Figure S1** CD8 immunohistochemistry staining in tumor samples. A cutoff value of 5% was used to group the samples. Left, below 5% (low ratio of CD8); Right, above 5% (high ratio of CD8).

**Figure S2** Apoptosis between different clinicopathological features. The distribution of apoptosis ratio was compared on different *EGFR* mutation subtypes (A and B), sex (C), age (D), smoking history (E), and disease stage (F). Low, medium and high levels of apoptosis were determined as <1%, 1–5% and ≥5%.

**Table S1** Logistic regression analysis of CD8 T cell apoptosis.
