# Supplementary figures and images for: Association of CD8 T cell apoptosis and EGFR mutation in non‐small lung cancer patients
Source: Thorac Cancer. 2020 Jun 4;11(8):2130–6. doi: 10.1111/1759-7714.13504 (PMC7396381; doi:10.1111/1759-7714.13504)

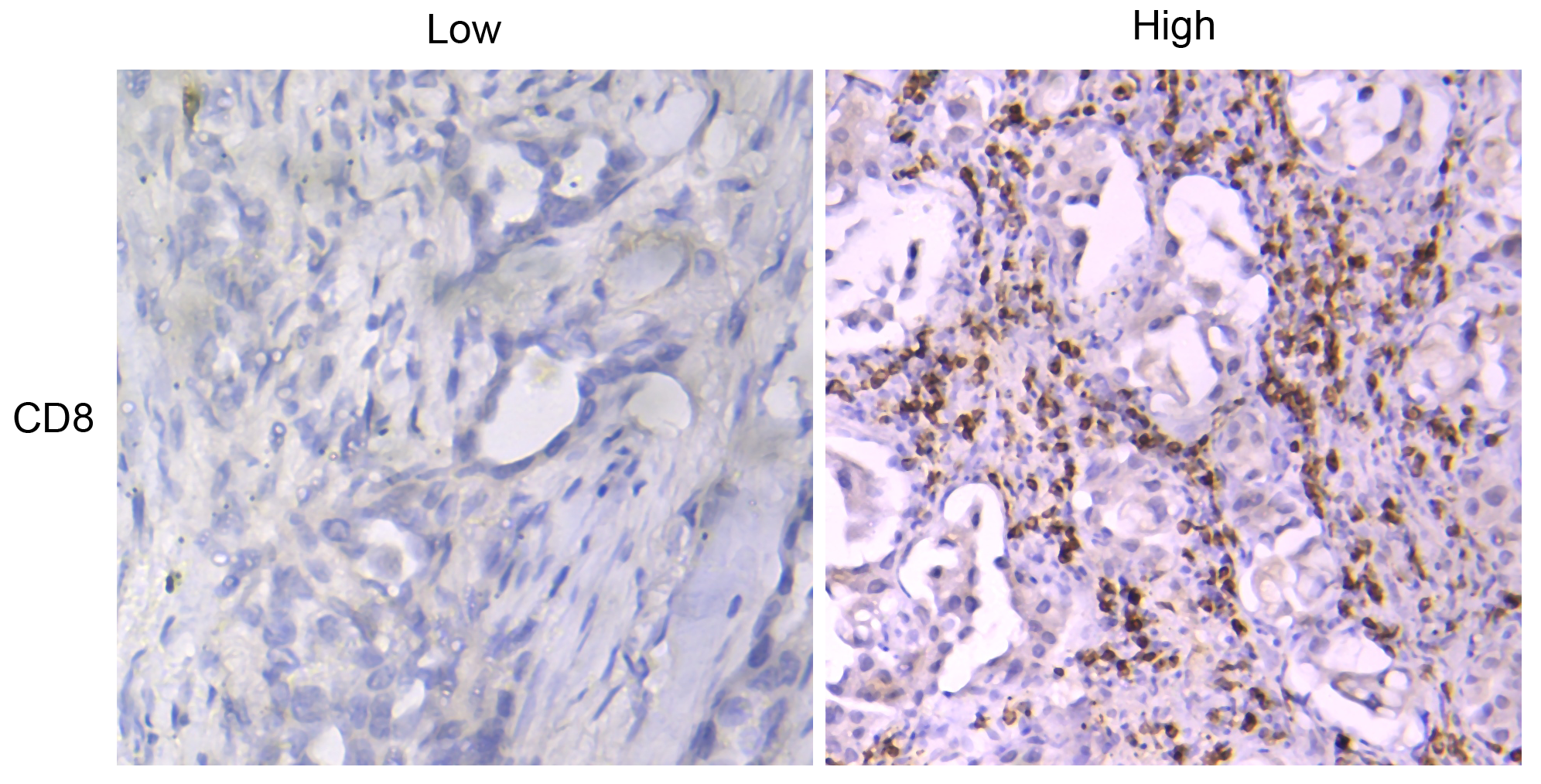

Supplement: Supplementary file 1 — Figure S1 CD8 immunohistochemistry staining in tumor samples. A cutoff value of 5% was used to group the samples. Left, below 5% (low ratio of CD8); Right, above 5% (high ratio of CD8). [file TCA-11-2130-s001.tif]

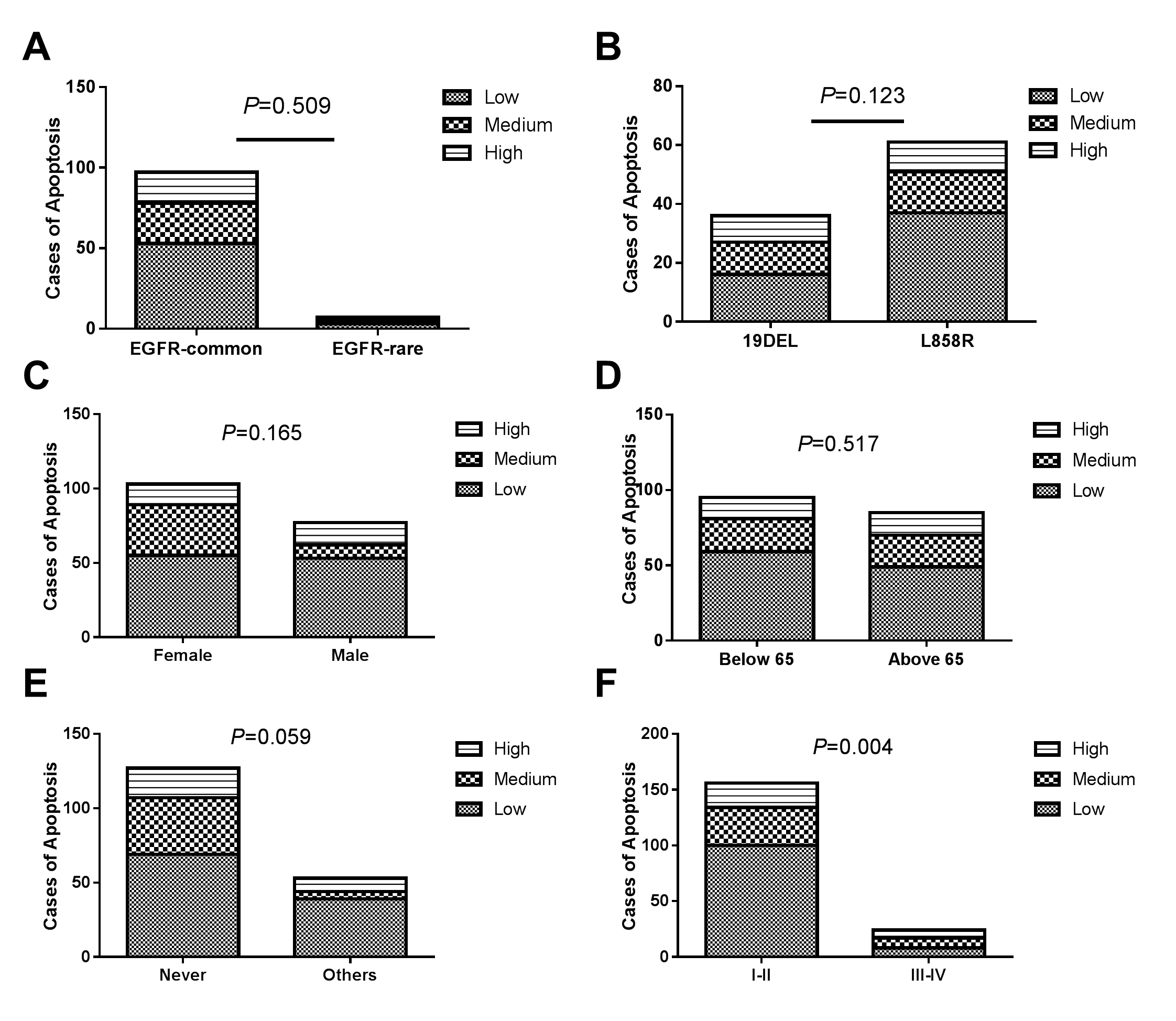

Supplement: Supplementary file 2 — Figure S2 Apoptosis between different clinicopathological features. The distribution of apoptosis ratio was compared on different EGFR mutation subtypes (A and B), sex (C), age (D), smoking history (E), and disease stage (F). Low, medium and high levels of apoptosis were determined as <1%, 1–5% and ≥5%. [file TCA-11-2130-s002.tif]
