# Supplementary material for: Association of CD8 T cell apoptosis and EGFR mutation in non‐small lung cancer patients
Source: Thorac Cancer. 2020 Jun 4;11(8):2130–6. doi: 10.1111/1759-7714.13504 (PMC7396381; doi:10.1111/1759-7714.13504)
Supplement: Supplementary file 3 — Table S1 Logistic regression analysis of CD8 T cell apoptosis. [file TCA-11-2130-s003.docx]

Supplementary Table 1. Logistic regression analysis of CD8 T cell apoptosis.

| Character | P |
| --- | --- |
| Sex | 0.103 |
| Age | 0.871 |
| Smoking status | 0.140 |
| Disease stage | 0.003 |
| EGFR status | 0.108 |
